# Serum CA724 has no diagnostic value for gastrointestinal tumors

**DOI:** 10.1007/s10238-023-01025-0

**Published:** 2023-03-15

**Authors:** Huiru Cao, Liuming Zhu, Lin Li, Wei Wang, Xiaoping Niu

**Affiliations:** 1 Laboratory of Digestion, Department of Gastroenterology, Yijishan Hospital of Wannan Medical College, Wuhu, Anhui Province People’s Republic of China; 2 Department of Gastroenterology, Yijishan Hospital of Wannan Medical College, No. 2, Zheshan Road, Wuhu, 241000 AnhuiProvince People’s Republic of China

**Keywords:** CEA, CA199, CA125, CA724, Gastrointestinal tumors

## Abstract

**Objective:**

This study aimed to explore the predictive values of serum carcinoembryonic antigen (CEA), carbohydrate antigen (CA) 199, CA125 and CA724 in the diagnosis of gastrointestinal tumors.

**Methods:**

Among patients treated for gastrointestinal tumors at the First Affiliated Hospital of Wannan Medical College between December 2020 and March 2022, 572 patients were reviewed as the tumor group, and 700 healthy subjects from the physical examination center of the same hospital were reviewed as the control group. We evaluated the correlation between serum CEA, CA199, CA125, CA724 levels and pathological features in 572 patients with gastrointestinal tumors.The levels of serum CEA, CA199, CA125 and CA724 were compared between the two groups, and the area under the receiver operating characteristic (ROC) curve (AUC) was used to evaluate the diagnostic efficacy of these markers alone and in combination.

**Results:**

Serum CEA level was correlated with tumor stage and metastasis, and CA199 was correlated with tumor stage, lymph node involvement and metastasis. CA125 and CA724 have no correlation with tumor pathological features. The levels of serum CEA, CA199 and CA125 were significantly increased in the tumor group compared with the control group, while serum CA724 levels did not significantly differ between groups (*p* > 0.05). In addition, in patients with gastric cancer (GC), esophageal cancer (EC), pancreatic cancer (PC), gallbladder cancer (GBC) or colorectal cancer (CRC), the serum CEA, CA199 and CA125 levels were significantly higher than those in the control group (*p* < 0.05). However, serum CA724 levels were increased only in CRC patients (*p* < 0.05). ROC curve evaluation results showed that while CA199, CA125 and CA724 alone had poor diagnostic efficacy in the tumor group, CEA was better. Specifically, CEA had better diagnostic efficacy in GC, PC, GBC and CRC; additionally, CA199 and CA125 had better diagnostic efficacy in PC. However, CA724 showed no diagnostic value in the tumor group and the single gastrointestinal tumor group. For diagnosis with multiple-marker combinations, CEA + CA199 + CA125 had the best diagnostic performance (AUC = 0.776, AUC = 0.650, AUC = 0.896, AUC = 0.840, AUC = 0.793) in the GC, EC, PC, GBC and CRC groups, and the sensitivity of multiple-marker combined detection was better than that of single-marker detection.

**Conclusions:**

Serum CA724 has no diagnostic value for gastrointestinal tumors, and it cannot evaluate the pathological status of tumors. Serum CEA has excellent diagnostic efficacy in GC, PC, GBC and CRC, and its expression level is related to tumor stage and metastasis. Additionally, CA199 and CA125 have good diagnostic efficacy in PC. Among them, CA199 level was related to tumor stage, lymph node involvement and metastasis, and CA125 level was not related to pathological status. In addition, the multiple-marker combination CEA + CA199 + CA125 has the best diagnostic efficacy in GC, EC, PC, GBC and CRC.

**Supplementary Information:**

The online version contains supplementary material available at 10.1007/s10238-023-01025-0.

## Introduction

Digestive system tumors are the most common form of tumors; they account for approximately 70% of all tumors [1] and are the leading cause of death in China. [2]. According to data from the 2020 Global Cancer Surveillance System, the spectrum of gastrointestinal tumors in China is changing; the incidence rates of gastric cancer (GC) and esophageal cancer (EC) are decreasing, and the incidence rates of colorectal cancer (CRC) and pancreatic cancer (PC) are increasing [3]. Gastrointestinal tumors have no specific manifestations in the early stage of disease, and the only effective screening and diagnosis method is endoscopy. Due to its invasive nature of treatment and relative lack of comfort in practice, patient compliance is poor. Most patients are willing to choose endoscopic diagnosis only when they have specific manifestations; however, most of the diagnostic results at that time indicate middle- and late-stage disease, resulting in difficult treatment, high cost, poor prognosis and an increased burden on patients and the social economy. Therefore, finding another safe, convenient and effective screening method for digestive system tumors is essential. Currently, tumor marker (TM) detection in blood is mostly used to screen early cancer worldwide [4]. In Japan, nine types of TMs are certified for use in tumor monitoring: carcinoembryonic antigen (CEA), carbohydrate antigen (CA) 199, CA125, CA50, CA724, sialyl Tn antigens (STN), alpha-fetoprotein (AFP), inhibitor of apoptosis (IAP) and tissue polypeptide antigen (TPA) [5]. In China, most physical examination centers have also included digestive system TMs in early cancer screening, mainly including CEA, CA199, CA125 and CA724. However, there is still a lack of clarity regarding the specific diagnostic value of these TMs in different types of gastrointestinal tumors. Some single indicators have low sensitivity, poor diagnostic efficiency or even no diagnostic efficiency, while combining multiple indicators can improve diagnostic efficacy and sensitivity [6, 7]. This evidence prompts us to use the combination of multiple indicators to diagnose early-stage gastrointestinal tumors. Here, we retrospectively analyzed the levels of serum TM CEA, CA199, CA125 and CA724 in patients with gastrointestinal tumors, including GC, EC, PC, GBC and CRC, and compared them with the data from patients in a healthy physical examination center. The correlation between gastrointestinal tumors pathological parameters and the evaluated CEA,CA199,CA125 and CA724 was also assessed.The area under the receiver operating characteristic (ROC) curve (AUC) was used to evaluate the diagnostic efficacy of the markers alone and in combination and to explore their predictive value in diagnosing gastrointestinal tumors.

## Materials and methods

### Subjects

This study was a retrospective study approved by the Ethics Committee of Yijishan Hospital of Wannan Medical College. Patients with gastrointestinal tumors admitted to Yijishan Hospital between December 2020 and March 2022 were systematically reviewed. The tumor group included 572 patients with gastrointestinal tumors and an average age of 63.22 ± 0.37 years, including 321 males and 251 females with ages ranging from approximately 35 to 85 years. The inclusion criterion was a pathologically confirmed diagnosis of gastrointestinal tumors; there were 193 patients with GC (the histological type was adenocarcinoma), 143 patients with EC (the histologic type was squamous cell carcinoma), 65 patients with PC (the histological type was ductal adenocarcinoma), 35 patients with GBC (the histological type was adenocarcinoma) and 151 patients with CRC (the histological type was adenocarcinoma). These clinicopathological characteristics were collected in this study including pathological tumor stage (pT stage), pathological node stage (pN stage), metastasis and differentiation. The patients were staged according to the TNM staging description (eighth edition) of the International Union Against Cancer (UICC)/American Cancer Society (AJCC).

The exclusion criteria were as follows: (1) previous history of malignant tumor treatment; (2) unclear diagnosis; and (3) incomplete data. A total of 700 healthy people who underwent physical examination in the physical examination center of our hospital during the same period were selected as the control group, including 280 males and 420 females with ages that ranged from approximately 35 to 83 years and an average age of 60.15 ± 0.65 years. There were no significant differences in age or sex between the tumor group and the control group (*p* > 0.05), making them comparable.

### Measurement of serum CEA, CA199, CA125 and CA724

Fasting venous blood (5 ml) was drawn in the morning in a procoagulant tube, left at room temperature for 30 min, and centrifuged at 3000 RPM/min for 10 min so that the upper serum could be collected. An AXSYMI2000 chemiluminescence analyzer (Abbott, USA) and its supporting reagents were used to detect serum CEA, CA199, CA125 and CA724 levels, and the reference ranges of each marker were as follows [8]: CEA 0–5 ng/ml, CA199 0–37 U/ml, CA125 0–35 U/ml and CA724 0–7 U/ml. The test result was judged as positive when it was greater than the reference range.

### Sensitivity and specificity

The formulas for calculation of sensitivity and specificity were as follows: sensitivity = number of true-positive cases/(number of true-positive cases + number of false-negative cases) × 100%; specificity = number of true-negative cases/(number of true-negative cases + number of false-positive cases) × 100%.

### ROC curve

The ROC curve was drawn based on serum tumor marker levels, and the AUC value was used to judge the diagnostic efficacy. When the AUC > 0.5, the closer the AUC was to 1, the better the diagnostic effect. When the AUC was between approximately 0.5 and 0.7, the diagnostic efficacy was poor; when the AUC was between 0.7 and 0.9, the diagnostic efficacy was good; and when the AUC was above 0.9, the diagnostic efficacy was excellent. When the AUC was ≤ 0.5, the diagnostic method was considered to be completely ineffective and deemed to have no diagnostic value.

### Statistical analysis

SPSS 18.0 (SPSS Inc., Chicago, IL, USA) and GraphPad Prism 7.0 (GraphPad Software, Inc., USA) were used for statistical analysis. The Chi-square test was used to identify associations between each TMs levels and clinicopathological features.The measurement data are expressed as the mean ± standard deviation (SD) and were analyzed by independent sample *t* tests. A *P* value < 0.05 was considered indicative of a significant difference, and an asterisk (*) was used to signify *p* < 0.05.

## Results

### Correlation between serum CEA, CA199, CA125, CA724 and clinicopathological features of gastrointestinal tumors

Correlation analysis showed that CEA levels have significant correlations with pT stage, metastasis and CA199 levels have significant correlations with pT stage, pN stage and metastasis(Tables 1,* p* < 0.05). However, serum CA125 and CA724 levels had no significant correlation with pT staege, pN stage, metastasis and differentiation status of gastrointestinal tumors (Tables 2,* p* > 0.05).

**Table 1:** The relationship of CEA, CA199 and clinicopathological feature of gastrointestinal tumors

Feature	Case	CEA				CA199			
		Normal	High	*χ*^2^	*P*	Normal	High	χ^2^	*P*
pT stage				4.551	0.033			8.549	0.004
T1-2	178	130	48			145	33		
T3-4	394	252	142			275	119		
pN stage				3.677	0.159			7.462	0.024
N0-1	177	129	48			138	39		
N2	259	173	96			194	65		
N3	136	90	46			88	48		
Metastasis				6.303	0.012			6.119	0.013
No	521	356	165			390	131		
Yes	51	26	25			30	21		
Differentiation				3.665	0.056			2.409	0.121
Poor	187	135	52			145	42		
Medium/high	385	247	138			275	110

**Table 2:** The relationship of CA125, CA724 and clinicopathological feature of gastrointestinal tumors

Feature	Case	CA125				CA724			
		Normal	High	χ^2^	*P*	Normal	High	χ^2^	*P*
pT stage				0.158	0.691			0.008	0.927
T1-2	178	140	38			129	49		
T3-4	394	304	90			287	107		
pN stage				4.375	0.112			1.133	0.568
N0-1	177	145	32			134	45		
N2	259	191	68			183	76		
N3	136	108	28			101	35		
Metastasis				0.247	0.619			0.129	0.719
No	521	403	118			380	141		
Yes	51	41	10			36	15		
Differentiation				2.816	0.093				
Poor	187	153	34			138	49	1.071	0.301
Medium/High	385	291	94			268	117		

### Comparison of serum TM levels between patients with gastrointestinal tumors and healthy subjects

**Fig. 1:**
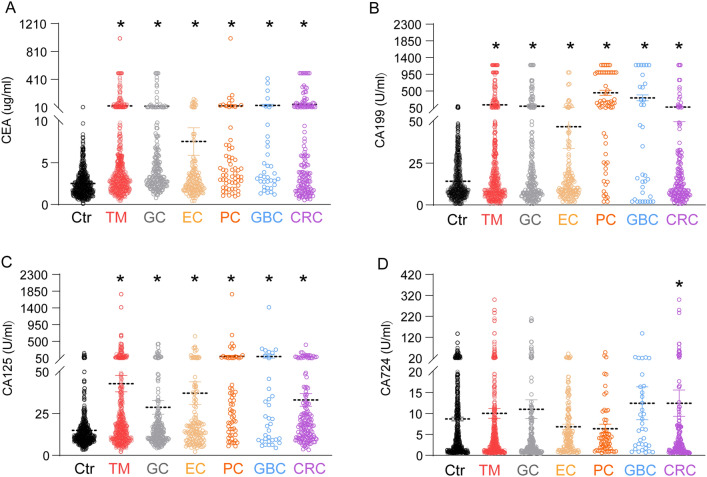
Comparison of serum TM levels between patients with gastrointestinal tumors and healthy physical subjects

To compare the diagnostic effects of different tumor markers in various digestive system tumors, we analyzed the TM data from different digestive system tumors. Overall, the levels of serum CEA, CA199 and CA125 were significantly increased in the tumor group compared to the control group (Fig. 1A-C *P*< 0.05), while CA724 levels showed no difference between patients in the tumor group and patients in the control group (Fig. 1D *P*> 0.05). In addition, the levels of serum CEA, CA199 and CA125 in patients with GC, EC, PC, GBC and CRC were higher than those found in the control group (Fig. 1A-C  *P*> 0.05), while CA724 was increased in CRC patients alone (Fig. 1D  *P*> 0.05). Collectively, these results indicate that a single serum TM has a good diagnostic effect in most tumors, except for CA724.

Serum tumor marker measurements of (A) CEA, (B) CA199, (C) CA125 and (D) CA724 in healthy or tumor patients. Data are presented as the mean ± standard deviation (SD). Independent sample *t* test for A-D, *p < 0.05.

### The diagnostic efficacy of single markers for gastrointestinal tumors

**Fig. 2:**
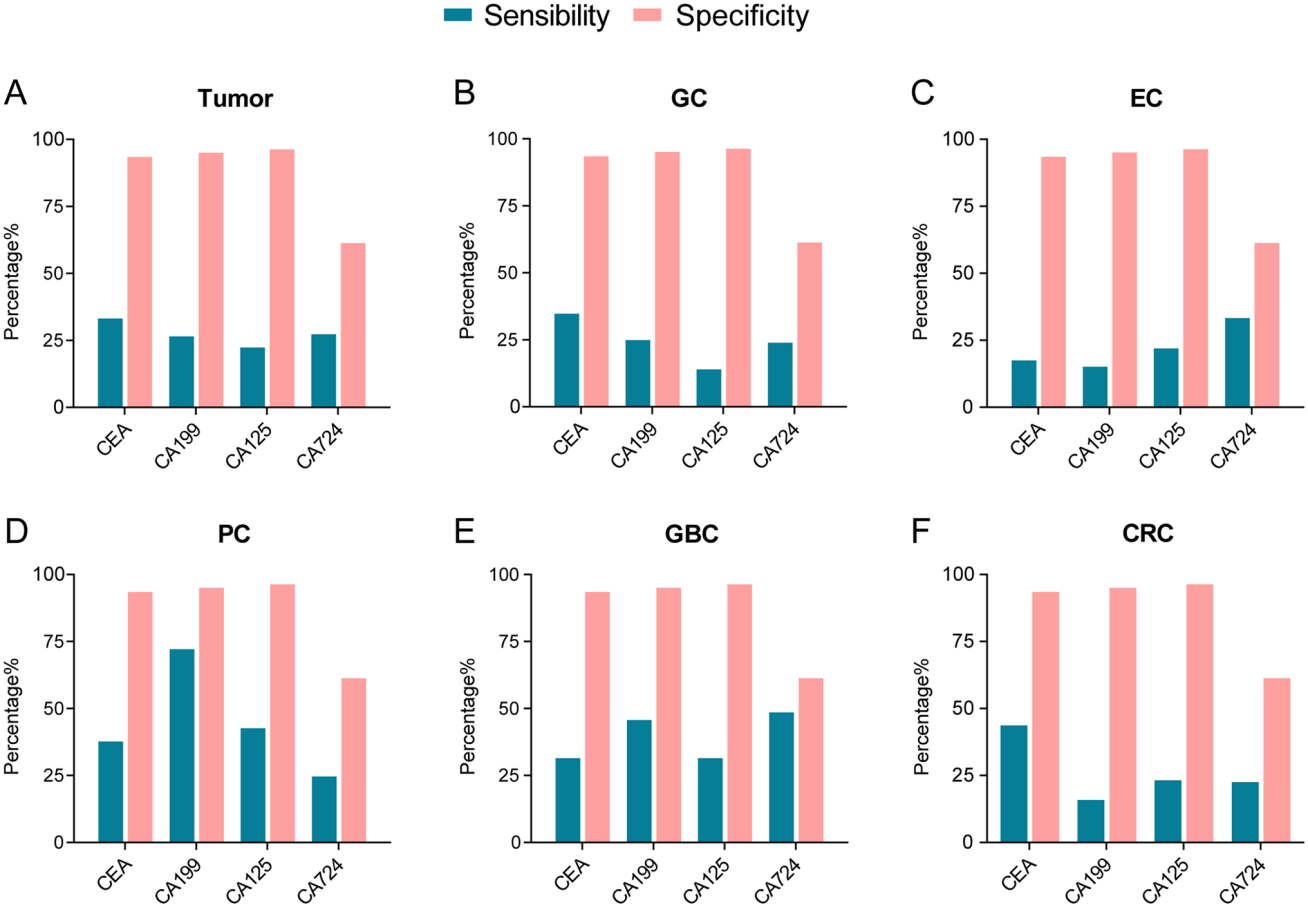
Specificity and sensitivity of tumor markers CEA, CA199, CA125 and CA724 in diagnostic efficacy regarding different types of gastrointestinal tumors

**Fig. 3:**
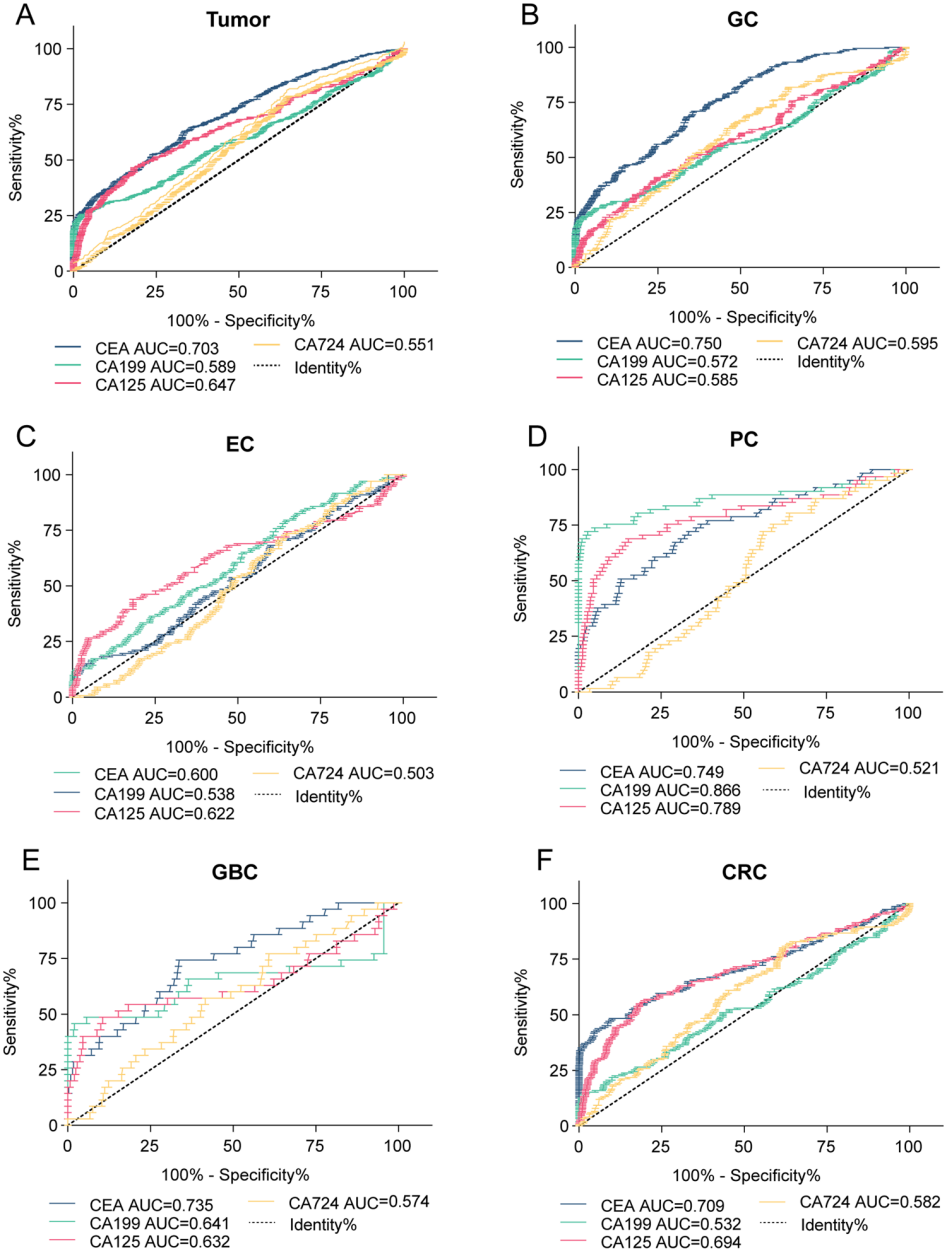
ROC curves of tumor markers CEA, CA199, CA125 and CA724 for the diagnosis of different types of gastrointestinal tumors

Next, we further analyzed the diagnostic efficacy of different single markers in various gastrointestinal tumors. In the tumor group, the sensitivity of CEA was 33.22%, the specificity was 93.43%, the AUC was 0.703, and the diagnostic efficacy was good. The sensitivity values of CA199, CA125 and CA724 were low, and the diagnostic efficacy for each marker was poor (Fig. 2A, 3A). The diagnostic performance of each marker in specific gastrointestinal tumors is summarized as follows: In GC patients, GBC and CRC, CEA had better diagnostic efficacy (AUC = 0.750, AUC = 0.735, AUC = 0.709), while CA199, CA125 and CA724 had poor diagnostic efficacy (Figs. 2B, E, F, and 3B, E, F) . In PC patients, CEA, CA199 and CA125 (AUC = 0.749, AUC = 0.866, AUC = 0.789) had better diagnostic performance, and CA199 had higher sensitivity and specificity (Figs. 2D, 3D). Together, these results showed that the diagnostic efficacy of CA724 was poor in both the overall tumor group and the single gastrointestinal tumor group (Fig. 2A–F, 3A–F).

Specificity and sensitivity of tumor markers of (A) tumor, (B) GC, (C) EC, (D) PC, (E) GBC and (F) CRC. Data are presented as the percentages (%).

ROC curves of tumor markers CEA, CA199, CA125 and CA724 are used for the diagnosis of (A) tumor, (B) GC, (C) EC, (D) PC, (E) GBC and (F) CRC. Data are presented as the AUC.

### The diagnostic efficacy of multiple markers in combination in gastrointestinal tumors

**Fig. 4:**
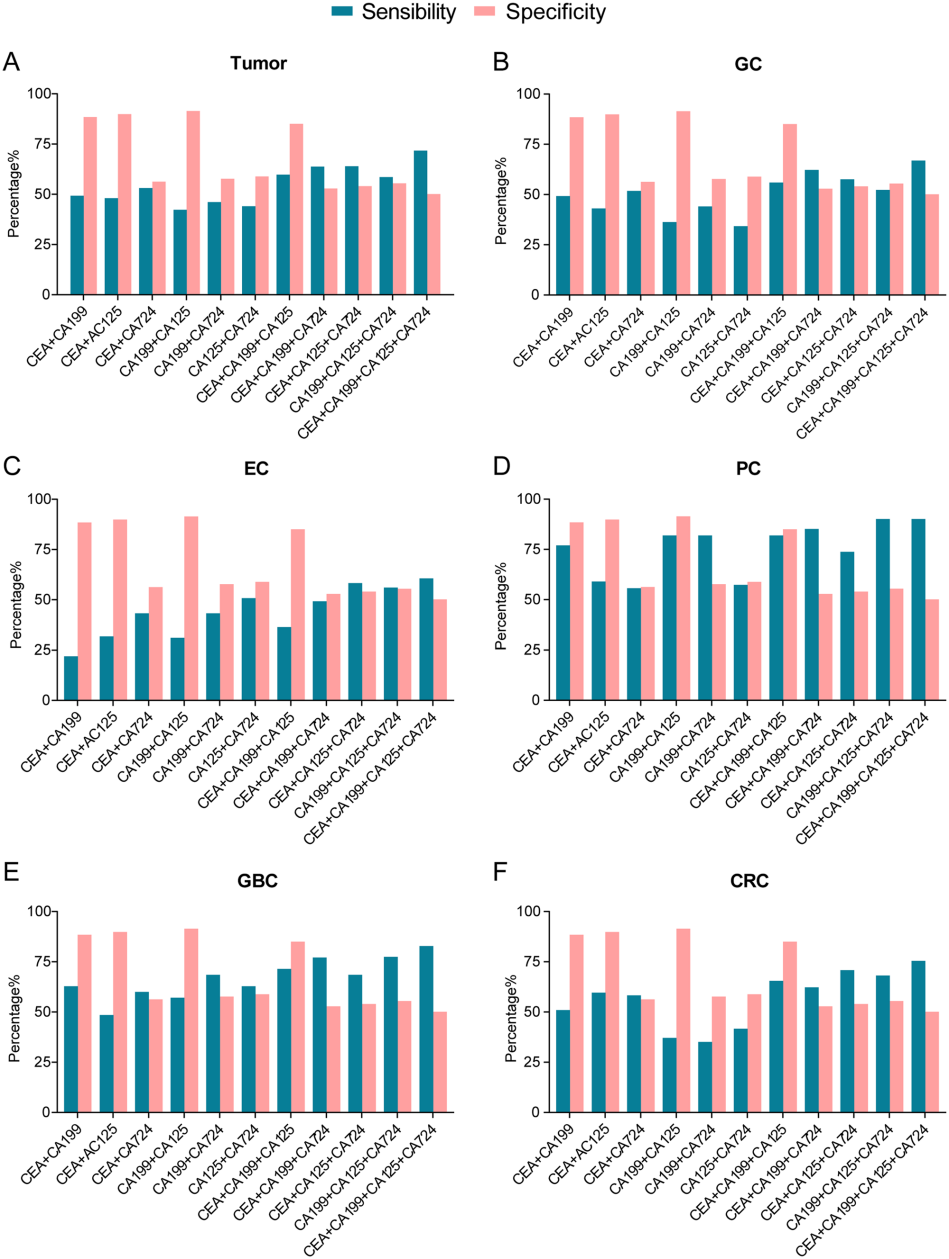
Specificity and sensitivity of multiple markers combined in the diagnosis of different types of gastrointestinal tumors

**Fig. 5:**
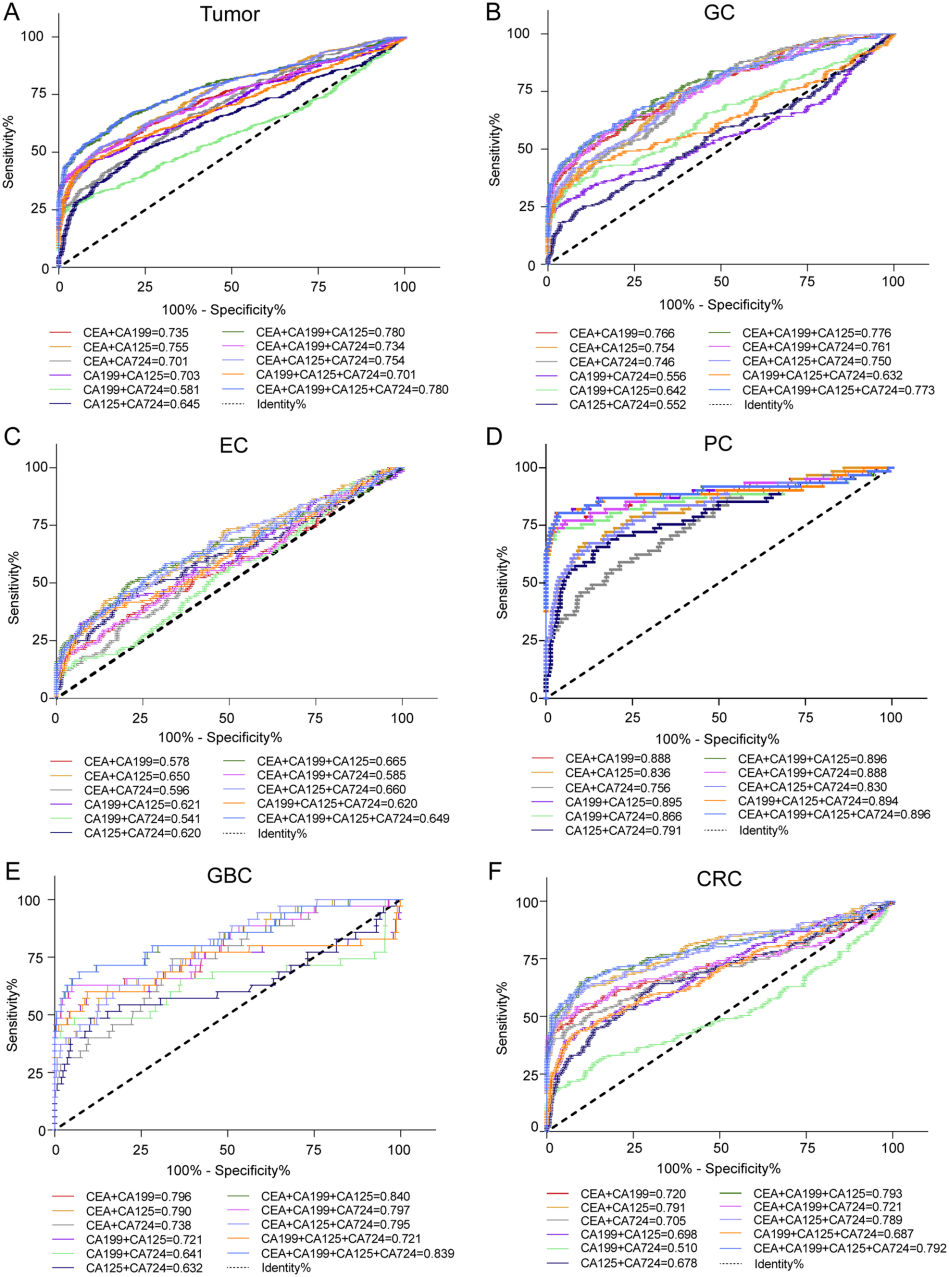
ROC curves of multiple-marker combined detection in the diagnosis of gastrointestinal tumors

Due to the low diagnostic efficacy of single tumor markers, we further explored diagnostic efficacy with different marker combinations. In this study, we explored different combinations of two, three or four of the markers CEA, CA199, CA125 and CA724 and observed that the diagnostic sensitivity of the multiple-marker combination was higher than that of a single marker, while the specificity decreased; the combination of CEA + CA199 + CA125 + CA724 had the highest sensitivity and the lowest specificity (Fig. 4A–F). The diagnostic efficacy of different combinations was as follows. The diagnostic efficacy of any combination for CEA, CA199 and CA125 in the tumor group for GC, EC, PC, GBC and CRC patients was higher than that of a single marker, and the combination of CEA + CA199 + CA125 had the best diagnostic efficacy (tumor: AUC = 0.780, GC: AUC = 0.776, EC: AUC = 0.650, PC: AUC = 0.896, GBC: AUC = 0.840, CRC: AUC = 0.793) (Fig. 5A–F). These results suggest that the diagnostic efficacy of the combination containing the tumor marker CA724 was slightly reduced.

Specificity and sensitivity of multiple markers combined in diagnosis of (A) tumor, (B) GC, (C) EC, (D) PC, (E) GBC and (F) CRC. Data are presented as the percentages (%).

ROC curves of multiple-marker combined detection in the diagnosis of (A) tumor, (B) GC, (C) EC, (D) PC, (E) GBC and (F) CRC. Data are presented as the AUC.

## Discussion

Cancer is still the leading cause of death in China. According to data from the 2020 Global Cancer Surveillance System [3], it is estimated that in China in 2022, 4.82 million people will be newly diagnosed with cancer, and 3.21 million people will die of cancer; 49.92% of cancer-related deaths will come from gastrointestinal tumors, and the prognosis for patients with such tumors is relatively poor. With the rapid development of China's social economy and people's pursuit of health, early cancer screening has become an important diagnostic method. The tumor markers CEA, AFP, CA199, CA125 and CA724 are widely used in the auxiliary diagnosis of digestive system tumors; among them, AFP has been widely recognized as a specific diagnostic marker for liver cancer [9, 10], and CEA has better diagnostic value in the diagnosis of digestive system tumors [4, 11]. However, the predictive values of CA199, CA125 and CA724 for gastrointestinal tumors remain unclear. In this study, we evaluated the correlation between serum CEA, CA199, CA125, CA724 levels and pathological features in gastrointestinal tumors. The analysis showed that CEA levels have significant correlations with pT stage, metastasis and CA199 levels have significant correlations with pT stage, pN stage, metastasis(*p* < 0.05). However,serum CA125 and CA724 levels had no significant correlation with pT staege, pN stage, metastasis and differentiation status of gastrointestinal tumors (*p* > 0.05). The levels of serum CEA, CA199, CA125 and CA724 were analyzed to compare diagnostic efficacy between patients with various gastrointestinal tumors and healthy people on physical examination.

The results showed that the levels of CEA, CA199 and CA125 in the overall tumor group were significantly higher than those in the control group (*p* < 0.05), suggesting that CEA, CA199 and CA125 have significance in the prediction of gastrointestinal tumors, but their levels of performance were different across various gastrointestinal tumors. The serum CA724 level was not significantly different between the overall tumor group and the control group (*p* > 0.05) but was slightly increased in CRC (*p* < 0.05), suggesting that CA724 may have a certain significance in the prediction of CRC.

We further evaluated the diagnostic efficacy of single and combined detection of the above TMs in different gastrointestinal tumors. Regarding single markers, CEA had better diagnostic efficacy in the tumor group, while CA199, CA125 and CA724 had poor diagnostic efficacy. CEA had better diagnostic efficacy in GC, PC, GBC and CRC patients, and CA199 and CA125 had better diagnostic efficacy in PC patients. The diagnostic efficacy of CA724 was poor alone and in combination with other markers. As one of the most common tumor markers, CEA is widely used in diagnosing various tumors, with high specificity for GC [4] and good applicability for malignant tumors of the digestive system. Numerous studies [11, 12] have found that serum CEA levels of patients with GC [13], PC [14] and CRC [15] are significantly higher than those of patients in the control group, and the diagnostic efficacy is good. Our results also suggested that CEA has good diagnostic value for gastrointestinal tumors. CA199 is an oligosaccharide tumor-associated antigen that has been found to have a high positive rate in gastrointestinal tumors [4] and the best sensitivity in diagnosing PC [16]. In this study, the sensitivity of CA199 in PC was 72.131% (AUC = 0.866), suggesting that the high expression of CA199 has a high predictive value for PC. CA125 is a glycoprotein complex that is highly expressed in various tumors, such as ovarian cancer, GC, PC, and CRC [17–20]. In this study, the expression of CA125 was increased in all gastrointestinal tumors, and the sensitivity of single-marker detection was not high. The diagnostic efficacy of CA125 for GC, EC, GBC and CRC was poor, suggesting that the predictive value of this marker for gastrointestinal tumors is only high for PC. CA724 is a high molecular weight glycoprotein. Studies have shown that CA724 is increased in the serum levels of patients with GC, CRC and breast cancer (BC) [21–24], and it has good diagnostic performance for gastrointestinal tumors [25]. However, other studies have found that CA724 is not only expressed in tumor tissues but also highly expressed in the serum of gout patients [26] and moderately expressed in normal tissues of secretory endometrium and transitional colon mucosa [27]; this indicates that CA724 is not the exclusive product of tumor cells, rendering the role of CA724 as a predictor of gastrointestinal tumors controversial. In this study, CA724 expression was only slightly increased in CRC patients (*p* < 0.05), and its diagnostic efficacy was poor (AUC = 0.582), with a sensitivity of 22.52% and a specificity of 61.29%. There was no significant difference in the expression of CA724 in other gastrointestinal tumors, suggesting that single-marker detection using CA724 has low predictive value for gastrointestinal tumors.

In addition to evaluating the performance of each single marker in detection, we also combined two or four tumor markers to find the optimal diagnostic combination. The results showed that the sensitivity of any combination was higher than that of a single marker, and the false-negative rate was reduced, which indicated that detection with multiple markers in combination could improve the positive detection rate of gastrointestinal tumors. The combination of CEA, CA199 and CA125 had the best diagnostic performance in GC (AUC = 0.776), EC (AUC = 0.650), PC (AUC = 0.896), GBC (AUC = 0.840) and CRC patients (AUC = 0.793). It is suggested that the combined detection of multiple markers has a better predictive effect in gastrointestinal tumors. In different combinations of combined detection, we found that although the sensitivity of CA724 was increased when it was combined with other markers, the specificity was significantly reduced, and the AUC was slightly reduced or unchanged, which further proves that serum CA724 is unsuitable for being used as a single marker or in combination with other markers for the diagnostic screening of gastrointestinal tumors.

In summary, our results support the conclusion that CEA has better diagnostic efficacy in GC, PC, GBC and CRC patients, and its expression level is related to tumor stage and metastasis; that CA199 and CA125 have better diagnostic efficacy in PC patients, but poor diagnostic efficacy for GC, EC, GBC and CRC; and that CA724 is expressed at a higher level in CRC patients but has low diagnostic efficacy. Among them, CA199 level was related to tumor stage, lymph node involvement and metastasis, and CA125 level and CA724 level were not related to pathological status. Combined detection with CEA + CA199 + CA125 has the best diagnostic efficacy for GC, EC, PC, GBC,and CRC patients. Because the diagnostic efficacy of CA724 is poor either in the overall tumor group or in the single gastrointestinal tumor group, it is not recommended as a tumor marker for gastrointestinal cancer screening.

### Supplementary Information

Below is the link to the electronic supplementary material.

Supplementary file1 (PDF 97 KB)
