# Supplementary material for: Serum CA724 has no diagnostic value for gastrointestinal tumors
Source: Clin Exp Med. 2023 Mar 15;23(6):2433–42. doi: 10.1007/s10238-023-01025-0 (PMC10543537; doi:10.1007/s10238-023-01025-0)
Supplement: Supplementary file 1 — Supplementary file1 (PDF 97 KB) [file 10238_2023_1025_MOESM1_ESM.pdf]

Supplementary material

Table 1 Diagnostic performance of single marker in gastrointestinal tumors

	Marker	Sensitivity	Specificity	AUC (95%CI)
**Tumor**	CEA	33.217%	93.429%	0.703（0.675~0.732）
	CA199	26.573%	95.000%	0.589（0.557~0.621）
	CA125	22.378%	96.286%	0.647（0.616~0.678）
	CA724	27.273%	61.286%	0.551（0.519~0.582）
**GC**	CEA	34.715%	93.429%	0.750（0.712~0.787）
	CA199	24.870%	95.000%	0.572（0.521~0.623）
	CA125	13.990%	96.286%	0.585（0.537~0.633）
	CA724	23.834%	61.286%	0.595（0.550~0.640）
**EC**	CEA	17.424%	93.429%	0.600（0.546~0.651）
	CA199	15.151%	95.000%	0.538（0.484~0.592）
	CA125	21.970%	96.286%	0.622（0.561~0.682）
	CA724	33.333%	61.286%	0.503（0.455~0.551）
**PC**	CEA	37.705%	93.429%	0.749（0.680~0.818）
	CA199	72.131%	95.000%	0.866（0.798~0.934）
	CA125	42.623%	96.286%	0.789（0.715~0.863）
	CA724	24.590%	61.286%	0.521（0.458~0.584）
**GBC**	CEA	31.429%	93.429%	0.735（0.649~0.821）
	CA199	45.714%	95.000%	0.641（0.508~0.774）
	CA125	31.429%	96.286%	0.632（0.508~0.757）
	CA724	48.571%	61.286%	0.574（0.481~0.666）
**CRC**	CEA	43.709%	93.429%	0.709（0.655~0.763）
	CA199	15.894%	95.000%	0.532（0.476~0.587）
	CA125	23.179%	96.286%	0.694（0.642~0.746）
	CA724	22.517%	61.286%	0.582（0.532~0.632）

Tumor: gastrointestinal tumor group; GC: gastric cancer group; EC: esophageal cancer group; PC: pancreatic cancer group; GBC: gallbladder cancer group; CRC: colorectal cancer group

Table 2 Diagnostic performance of combined detection of multiple markers in the tumor group

Markers	Sensitivity	Specificity	AUC（95%CI）
CEA+CA199	49.301%	88.429%	0.735（0.707~0.764）
CEA+CA125	48.077%	89.857%	0.755（0.729~0.782）
CEA+CA724	53.147%	56.286%	0.701（0.673~0.730）
CA199+CA125	42.307%	91.429%	0.703（0.673~0.733）
CA199+CA724	46.154%	57.714%	0.581（0.548~0.613）
CA125+CA724	44.056%	58.857%	0.645（0.614~0.677）
CEA+CA199+CA125	59.790%	85.000%	0.780（0.754~0.807）
CEA+CA199+CA724	63.811%	52.857%	0.734（0.705~0.762）
CEA+CA125+CA724	63.986%	54.000%	0.754（0.727~0.781）
CA199+CA125+CA724	58.566%	55.429%	0.701（0.671~0.731）
CEA+CA199+CA125+CA724	71.678%	50.143%	0.780（0.752~0.806）

Table 3 Diagnostic performance of combined detection of multiple markers in GC

Markers	Sensitivity	Specificity	AUC（95%CI）
CEA+CA199	49.223%	88.429%	0.766（0.725~0.806）
CEA+CA125	43.005%	89.857%	0.754（0.715~0.792）
CEA+CA724	51.813%	56.286%	0.746（0.707~0.784）
CA199+CA125	36.269%	91.429%	0.642（0.592~0.691）
CA199+CA724	44.041%	57.714%	0.556（0.502~0.610）
CA125+CA724	34.197%	58.857%	0.552（0.502~0.603）
CEA+CA199+CA125	55.959%	85.000%	0.776（0.736~0.816）
CEA+CA199+CA724	62.176%	52.857%	0.761（0.720~0.802）
CEA+CA125+CA724	57.513%	54.000%	0.750（0.711~0.790）
CA199+CA125+CA724	52.332%	55.429%	0.632（0.581~0.684）
CEA+CA199+CA125+CA724	66.839%	50.143%	0.773（0.732~0.815）

Table 4 Diagnostic performance of combined detection of multiple markers in EC

Markers	Sensitivity	Specificity	AUC（95%CI）
CEA+CA199	21.970%	88.429%	0.578（0.520~0.637）
CEA+CA125	31.818%	89.857%	0.650（0.592~0.709）

CEA+CA724	43.182%	56.286%	0. 596（0.542~0.649）
CA199+CA125	31.061%	91.429%	0.621（0.563~0.679）
CA199+CA724	43.182%	57.714%	0.541（0.488~0.596）
CA125+CA724	50.758%	58.857%	0.620（0.562~0.677）
CEA+CA199+CA125	36.364%	85.000%	0.665（0.611~0.720）
CEA+CA199+CA724	49.242%	52.857%	0.585（0.528~0.643）
CEA+CA125+CA724	58.333%	54.000%	0.660（0.605~0.715）
CA199+CA125+CA724	56.061%	55.429%	0.620（0.560~0.674）
CEA+CA199+CA125+CA724	60.606%	50.143%	0.649（0.591~0.707）

Table 5 Diagnostic performance of combined detection of multiple markers in PC

Markers	Sensitivity	Specificity	AUC（95%CI）
CEA+CA199	77.049%	88.429%	0.888（0.827~0.949）
CEA+CA125	59.016%	89.857%	0.836（0.775~0.896）
CEA+CA724	55.738%	56.286%	0.756（0.689~0.822）
CA199+CA125	81.967%	91.429%	0. 895（0.832~0.958）
CA199+CA724	81.967%	57.714%	0.866（0.797~0.934）
CA125+CA724	57.377%	58.857%	0. 791（0.721~0.861）
CEA+CA199+CA125	81.967%	85.000%	0.896（0.832~0.959）
CEA+CA199+CA724	85.246%	52.857%	0.888（0.826~0.949）
CEA+CA125+CA724	73.770%	54.000%	0.830（0.766~0.894）
CA199+CA125+CA724	90.164%	55.429%	0.894（0.831~0.957）
CEA+CA199+CA125+CA724	90.164%	50.143%	0.896（0.833~0.960）

Table 6 Diagnostic performance of combined detection of multiple markers in GBC

Markers	Sensitivity	Specificity	AUC（95%CI）
CEA+CA199	62.857%	88.429%	0.796（0.702~0.890）
CEA+CA125	48.571%	89.857%	0.790（0.710~0.870）
CEA+CA724	60.000%	56.286%	0.738（0.653~0.822）
CA199+CA125	57.143%	91.429%	0.721（0.596~0.846）
CA199+CA724	68.571%	57.714%	0.641（0.508~0.774）
CA125+CA724	62.857%	58.857%	0.632（0.507~0.757）
CEA+CA199+CA125	71.429%	85.000%	0.840（0.756~0.924）
CEA+CA199+CA724	77.143%	52.857%	0.797（0.705~0.890）

CEA+CA125+CA724	68.571%	54.000%	0.795（0.719~0.871）
CA199+CA125+CA724	77.143%	55.429%	0.721（0.596~0.847）
CEA+CA199+CA125+CA724	82.857%	50.143%	0.839（0.754~0.924）

Table7 Diagnostic performance of combined detection of multiple markers in CRC

**Markers**	**Sensitivity**	**Specificity**	**AUC（95%CI）**
CEA+CA199	50.993%	88.429%	0.720（0.663~0.776）
CEA+CA125	59.603%	89.857%	0.791（0.743~0.839）
CEA+CA724	58.278%	56.286%	0.705（0.650~0.761）
CA199+CA125	37.086%	91.429%	0. 698（0.646~0.749）
CA199+CA724	35.099%	57.714%	0.510（0.450~0.569）
CA125+CA724	41.722%	58.857%	0. 678（0.625~0.731）
CEA+CA199+CA125	65.563%	85.000%	0.793（0.743~0.843）
CEA+CA199+CA724	62.252%	52.857%	0.721（0.663~0.779）
CEA+CA125+CA724	70.861%	54.000%	0.789（0.740~0.837）
CA199+CA125+CA724	68.212%	55.429%	0.687（0.633~0.741）
CEA+CA199+CA125+CA724	75.497%	50.143%	0.792（0.741~0.842）
